# Systematically false positives in early warning signal analysis

**DOI:** 10.1371/journal.pone.0211072

**Published:** 2019-02-06

**Authors:** Georg Jäger, Manfred Füllsack

**Affiliations:** Institute of Systems Sciences, Innovation and Sustainability Research, University of Graz, Graz, Austria; University of Hyogo, JAPAN

## Abstract

Many systems in various scientific fields like medicine, ecology, economics or climate science exhibit so-called critical transitions, through which a system abruptly changes from one state to a different state. Typical examples are epileptic seizures, changes in the climate system or catastrophic shifts in ecosystems. In order to predict imminent critical transitions, a mathematical apparatus called early warning signals has been developed and this method is used successfully in many scientific areas. However, not all critical transitions can be detected by this approach (false negative) and the appearance of early warning signals does not necessarily proof that a critical transition is imminent (false positive). Furthermore, there are whole classes of systems that always show early warning signals, even though they do not feature critical transitions. In this study we identify such classes in order to provide a safeguard against a misinterpretation of the results of an early warning signal analysis of such systems. Furthermore, we discuss strategies to avoid such systematic false positives and test our theoretical insights by applying them to real world data.

## Introduction

Critical transitions are an important feature in many systems, in which the state of a system changes abruptly and a return to the previous state becomes difficult or even impossible. Such transitions can be observed in medicine [1–3], climate science [4, 5], economics [6] and ecology [7–9].

For each of those systems it is important to predict an imminent critical transition in order to prevent it or adapt to it in time [10]. A prominent approach to this problem is to analyze time series. In this context, a time series is defined as a discrete-time series of data points in time order. Examples of such time-series are the average global temperature each year, the current value of a certain stock each day or brain activity each second. Such time series are then analyzed for statistical anomalies or similar markers that hint at a change on the micro-scale that is not yet visible on the macro-scale. Such markers are called early warning signals (EWSs) [11]. EWS analysis can be performed on measured data [12], data coming from equation-based simulations [13] and also data originating from agent-based models [14].

Unfortunately, the detection of such EWSs is not always straightforward. Sometimes the signals are hidden or obscured by topological effects [15] or the system shows a total lack of EWSs, despite featuring critical transitions [16]. This is called a false negative, since no signal can be detected, even though a critical transition is immanent. On the other hand, it is also possible to find EWSs in systems that will not show a critical transition. This is called a false positive and is especially problematic when used on statistical data in order to predict critical transitions in the future. In that case it is impossible to differentiate between a true and a false positive, since no data from future points in time are available to verify whether a critical transition follows the EWS or not. Various reasons can be responsible for the appearance of false positives: They can appear randomly, they can be caused by some microscopic system behavior that is not yet fully understood and sometimes the appearance of false positives is an inherent property of the system itself. Naturally, the last case is the most problematic one, because it means that such systems are unfit for traditional EWS analysis and one would need a different method to be able to predict critical transitions. In this paper we identify the classes of systems which are susceptible for such systematically false positives and find ways to overcome this challenge.

This paper is organized as follows: The section “Methods” gives a short introduction into the mathematical apparatus of EWS. Additionally, the investigated systems are introduced. Finally, possible solutions to the problem of systematic false positives are discussed. The section “Results” reports on the discovered EWS and showcases some of the performed EWS analyses in more detail. Also the effect of the proposed solutions is investigated. Furthermore, we test our findings by analyzing real world data of the global surface temperature change. In the “Discussion” section we comment on the relevance of the gained insights and how they can be used to improve state-of-the-art detection methods for critical transition, but also on the limitations of this study. Numeric details on the investigated systems can be found in Appendix A.

## Methods

### Early warning signals

Many systems have a so-called attractive stable state, i.e. a state that the system does not leave, even under small perturbations [17]. Most complex systems have several such states and due to internal or external dynamics, the system can change from one stable state to a different stable state. When this change is sudden, the transition is called a critical transition [18]. The most interesting point during such a transition is the tipping point, where the system is influenced by the attraction of two ore more stable states and could evolve into either one. Even though the proximity to a tipping point is not sufficient for a critical transition to occur (the system could as well bounce back to its original state) it is a necessary requirement for a critical transition. In order to identify if a system is close to such a tipping point, EWS analysis can be used.

Systems close to a tipping point show slightly different behavior than systems far away from it. When perturbed their recovery time increases: They need longer to evolve back to their original state. This effect is called critical slowing down (CSD) [19]. CSD leaves different statistical markers that can be detected by EWS analysis. On the one hand, the variance of the data increases, which is a direct effect of the increased recovery time: If the system needs more time to return to its stable state, it will spend more time away from this state, increasing the variance when compared to a system that instantly returns to its stable state. Furthermore, the slow recovery also leads to a higher autocorrelation. Systems close to a tipping point can also become less symmetric, which shows in the skewness of a time series. Skewness can be seen as a measure of the degree of asymmetry of a given distribution. In the context of EWS, the analyzed data points do not originate from a distribution, but are small sections of a given time series [20]. Nevertheless, calculating skewness is done in the usual way, see Eq (3). Also single values that are further away from the mean become more frequent near a tipping point. This phenomenon is called flickering [21] and can be detected in the kurtosis [22] of a time series.

In order to perform an EWS analysis we will look at the following four properties of a time series:

- Standard deviation *SD*
- Autocorrelation at lag 1 *AC*^(1)^
- Skewness *S*
- Kurtosis *K*

For the time series *X* containing *N* elements labeled *x*_*i*_, these properties are calculated using the following equations:

$$\begin{array}{c} \sigma (X)=\sqrt{\frac{\sum_{i=1}^{N}{\left(x_{i}-\bar{x}\right)}^{2}}{N-1}} \end{array}$$ (1)

$$\begin{array}{c} AC^{\left(1\right)}\left(X\right)=\frac{1}{N-1}\sum_{i=1}^{N}(\frac{x_{i}-\bar{x}}{\sigma_{x}})(\frac{y_{i}-\bar{y}}{\sigma_{y}}) \end{array}$$ (2)

$$\begin{array}{c} S(X)=\frac{\frac{1}{N}\sum_{i=1}^{N}{\left(x_{i}-\bar{x}\right)}^{3}}{{\sqrt{\frac{1}{N-1}\sum_{i=1}^{N}{\left(x_{i}-\bar{x}\right)}^{2}}}^{\;3}} \end{array}$$ (3)

$$\begin{array}{c} K(X)=\frac{\frac{1}{N}\sum_{i=1}^{N}{\left(x_{i}-\bar{x}\right)}^{4}}{{\left(\frac{1}{N}\sum_{i=1}^{N}{\left(x_{i}-\bar{x}\right)}^{2}\right)}^{2}}-3 \end{array}$$ (4)

Here $\bar{x}$ denotes the mean of *x* and *y*_*i*_ are the elements of a time series that is generated by shifting X by 1, so to that *y*_*i*_ = *x*_*i*−1_.

### Investigated systems

In order to generate results that are not specific to individual systems, but rather universal properties of EWS analysis, we focus our investigation on generic systems. To cover a broad range of possible applications we simulate the most common forms of growth and decay. An overview of the investigated systems is given in in Table 1. Numerical details on how the time series were produced can be found in Appendix A. By this choice of example functions, many common systems can be described. Linear systems are, for example, often encountered in ecology and economics [23, 24]. Economic growth and population growth can be described as an exponential growth, as long as we are far away from the maximal capacity [25, 26] or with a logistic function when the effect of the maximal capacity becomes relevant [27]. Also the more exotic investigated functions, like the trigonometric ones, have scientific applications. The solar cycle [28] as well as many climate oscillations [29, 30] are sine-like. Although not all systems of interest to EWS analysis can be described or approximated by the chosen systems or linear combinations thereof, we hope that essential features identified for these generic systems can also be translated to specific ones.

**Table 1:** Investigated systems and their abbreviations.

	linear	quadratic	exponential	logarithmic	trigonometric	logistic
growth	LG	QG	EG	LNG	TG	LOG
decay	LD	QD	ED	LND	TD	LOD

Note, that by design none of the investigated systems are capable of having critical transitions. That way the distinction between false positives and correct positives becomes trivial, since all detected EWS must be false positives. This property makes the systems ideal for identifying systematically false positives.

All systems are simulated with statistical noise, so that they resemble real measurements or simulations more closely. This noise can be absolute (i.e. independent of the value *x*_*i*_) or relative to *x*_*i*_. We produce time series of 10000 time steps and calculate the previously defined properties relevant for EWS analysis in rolling windows of size 500. Then we search for significant EWSs: an increase in both *σ* and *AC*^(1)^, a change in *S* or an increase in *K*. The simulations were performed using Python. After initialization of each system their time development is calculated from the equations given in Appendix A. As a next step artificial noise is added. For absolute noise a Gaussian-distributed random number is added, while for relative noise each data point is multiplied by a Gaussian-distributed random number. Details on the random numbers are also given in Appendix A. The generated time series can be downloaded from https://figshare.com/articles/falseEWS_rar/7472099.

### Scaling and detrending

In systems that show systematic false positives we look at strategies for removing those signals. Different approaches are thinkable and it is not a priori clear which are suitable and which are not. We use methods related to scaling and detrending, commonly used in data preparation [31–34]. The effect of five distinct methods is investigated:

- Local scaling
- Global scaling
- Detrending by local first-order polynomial fits
- Detrending by global higher-order polynomial fits
- Detrending by moving averages

For local scaling, we calculate the mean of every rolling window and subtract it from every data point. That way, the average value of each rolling window is zero, thus the absolute value of the original data point has no influence on the EWS analysis, possibly reducing the chance for false positives.

Global scaling is not done for each rolling window individually, but for the investigated time series as a whole. In addition to subtracting the global average, we also use a multiplicative factor on the whole time series to fix the standard deviation to 1. This can only be done on a global scale, since scaling the data for each rolling window individually would eliminate any EWS originating from standard deviation.

One possible way for detrending is to calculate a linear fit for the data of each rolling window using linear regression [35, 36]. We then subtract this fit from the data to find the residuals. These residuals are then the input signals for the EWS analysis. In addition to setting the mean of each rolling window to 0, this process also filters out macroscopic linear trends, so that the investigated data is more directly related to the micro-scale. Note, that this process only filters out the linear trend. This means that higher order trends remain in the detrended data and can be analyzed. In this sense, linear regression is not performed for the usual reason: Traditionally, linear regression is used to approximate a time series by a linear function as best as possible. Here on the other hand, the regression line is subtracted from the data. Thus the goal is not to approximate the data, but to remove the linear part to focus on the nonlinear effects.

A different approach to detrending works on a global scale, i.e. the whole time series, not just the investigated window. We calculate a fit using a 5th order polynomial. This order is high enough to satisfyingly describe the investigated systems. Subtracting this global fit should completely filter out macroscopic changes of the system and thus reduce false positives in EWS analysis.

The computationally inexpensive, and therefore most prominent way, of detrending data is using moving averages. For this technique the average value of the previous *x* time steps is subtracted from each data point. It is also possible to define an exponential weight factor, to increase the weight of closer data points. In any case, subtracting the moving average removes the macroscopic trend of a time series and we can investigate microscopic fluctuations in the form of residuals. For this study, we used *x* = 50, but the actual value of *x* does not change whether an EWS can be detected or not, but only influences the smoothness of the data and resulting signals.

## Results

**Fig 1:**
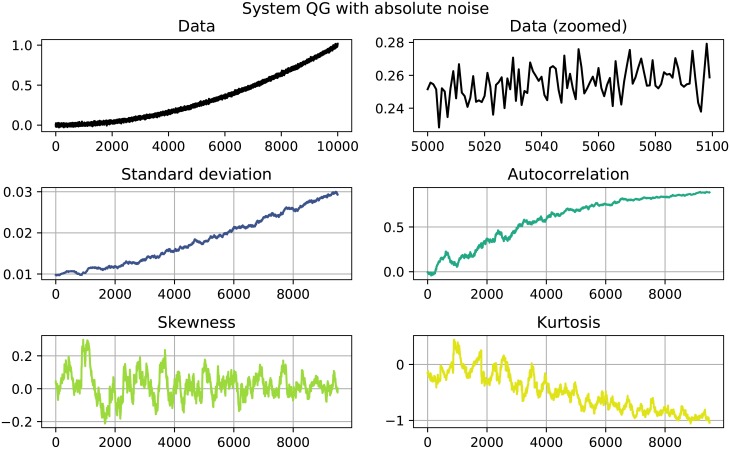
System QG with absolute noise. Both standard deviation (center row, left) and autocorrelation (center row, right) show a significant positive trend.

**Fig 2:**
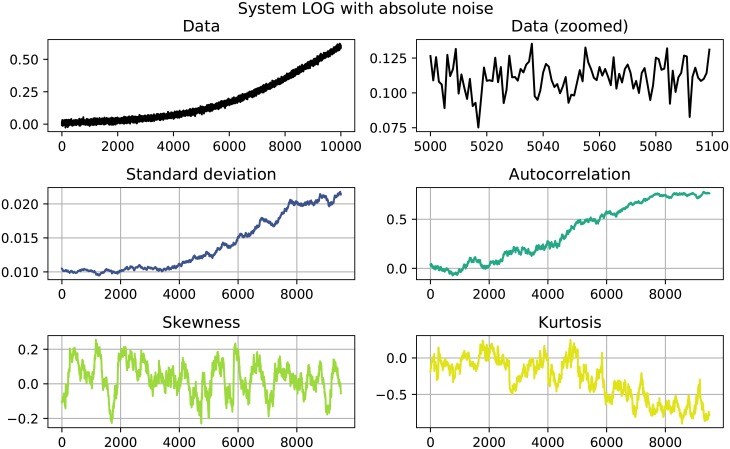
System LOG absolute noise. Both standard deviation and autocorrelation show a significant positive trend.

**Fig 3:**
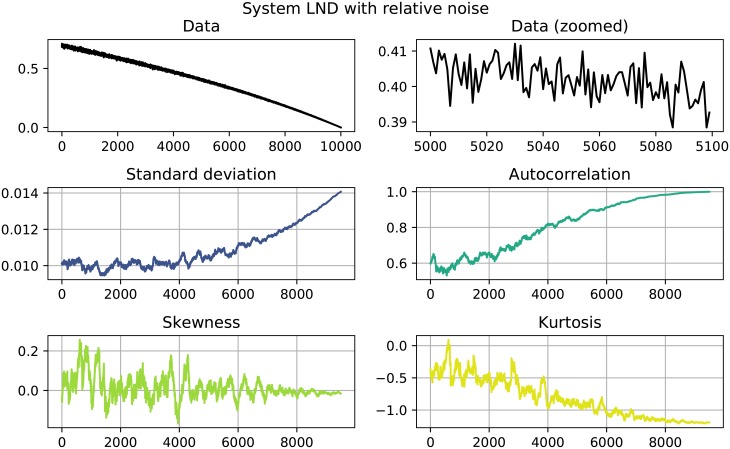
System LND with relative noise. Standard deviation shows a sharp increase, while autocorrelation increases more slowly.

**Fig 4:**
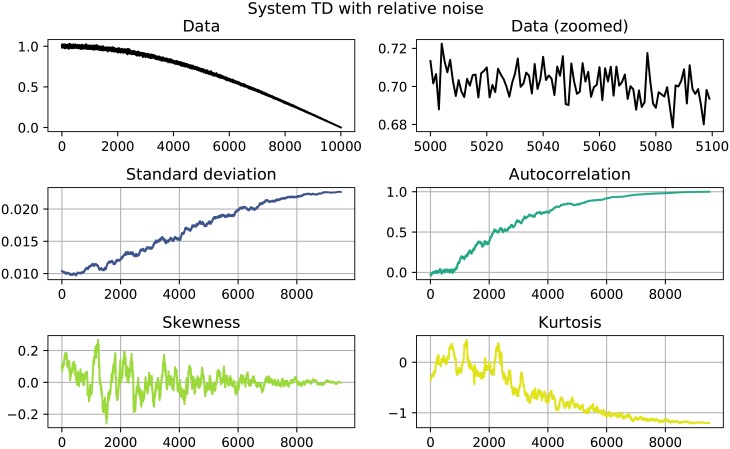
System TD with relative noise. Both standard deviation and autocorrelation show a significant positive trend.

**Fig 5:**
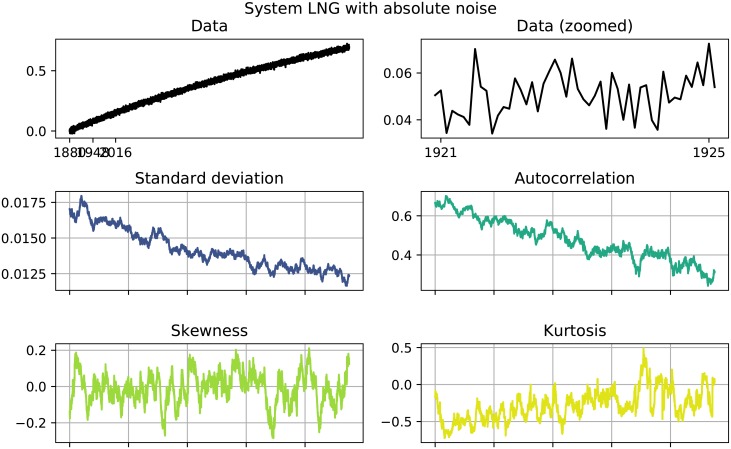
System LNG with absolute noise. For this system no EWS were detected. Standard deviation as well as autocorrelation decrease over time, while skewness and kurtosis show random fluctuations, but no clear trend.

The results of the performed EWS analysis are shown in Tables 2 and 3. Systems in which both *σ* as well as *AC*^(1)^ increase are highlighted, since this behavior could be interpreted as an EWS. Of the 22 investigated systems 11 show clear signals, namely the systems quadratic growth (QG), exponential growth (EG) and logistic growth (LOG) with absolute noise and the systems quadratic decay (QD), logarithmic decay (LND), trigonometric decay (TD) and logistic decay (LOD) with both absolute as well as relative noise. Here, a clear signals is constituted by a simultaneous increase in both *σ* and AC^(1)^ that goes beyond the usual fluctuation and stays that high until the end of the time series. Figs 1 to 4 show detailed results for some of the systems that showed EWS. A counterexample is given in Fig 5, where no EWS was detected. The first panel of those plots always shows the complete time series that was analyzed. The second panels zooms in on a smaller part of the data, in order to visualize finer structures and the random fluctuations. In the panels below, one can see the four analyzed properties of the time series: standard deviation, autocorrelation, skewness and kurtosis, calculated as detailed in Eqs (1) to (4).

**Table 2:** EWSs for systems with absolute noise.

System	*σ*	*AC*^(1)^	*S*	*K*
LG	no	no	no	no
QG	yes	yes	no	no
EG	yes	yes	no	no
LNG	no	no	no	no
TG	no	no	no	no
LOG	yes	yes	no	no
LD	no	no	no	no
QD	yes	yes	no	no
ED	no	no	no	no
LND	yes	yes	no	no
TD	yes	yes	no	no
LOD	yes	yes	no	no

**Table 3:** EWSs for systems with relative noise.

System	*σ*	*AC*^(1)^	*S*	*K*
LG	yes	no	no	yes
QG	yes	no	no	yes
EG	yes	no	no	no
LNG	no	no	no	yes
TG	no	no	no	yes
LOG	yes	no	no	yes
LD	no	yes	no	no
QD	yes	yes	no	no
ED	no	no	no	no
LND	yes	yes	no	no
TD	yes	yes	no	no
LOD	yes	yes	no	no

**Fig 6:**
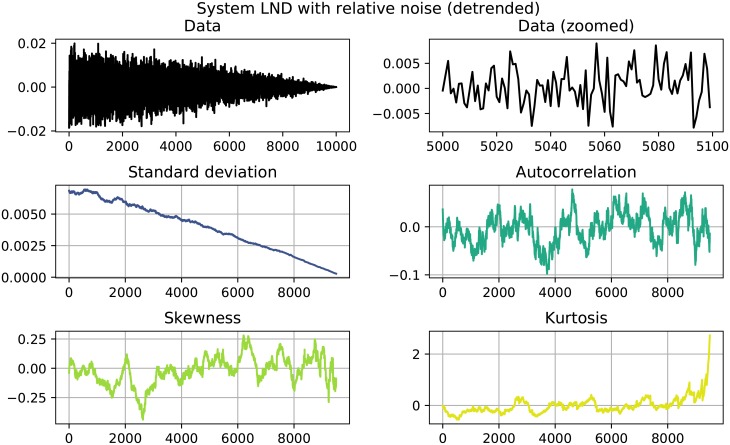
System LOD with relative noise after global detrending. Detrending successfully removes the false positive: The standard deviation now decreases, while the autocorrelation only shows random fluctuations.

In order to filter out the obtained false positives, we tried scaling and detrending with a local as well as with a global scope. Scaling, both local and global, did not result in the desired effect: all systems that showed false positives originally also showed them after scaling. Detrending, however, was well suited to remove false positives. An example can be seen in Fig 6, where the data from system LOD that showed false EWSs in the previous analysis, shows no EWSs after detrending. The qualitative result did not depend on the chosen method for detrending: All residuals were completely free of false positives for all investigated systems.

### Application to real-world data

In order to test the insights we obtained from investigating abstract systems, we now perform an EWS analysis on real-world data. As an example time series we use the global surface temperature change. Statistical data was provided by the Goddard Institute for Space Studies Surface Temperature Analysis (GISTEMP) [37]. From looking at the raw data, we see that the temperature change has absolute fluctuations of roughly 0.25 °C. We also detect an increase in temperature change that is similar to a quadratic growth. Thus, the time series resembles the previously investigated system QG. Because of these similarities between the temperature change data and one of the investigated systems, we expect that also the temperature change data behave similar to the system QG, i.e. we suspect that an EWS analysis of the raw data will show an increase in autocorrelation as well as an increase in standard deviation (see Table 2).

**Fig 7:**
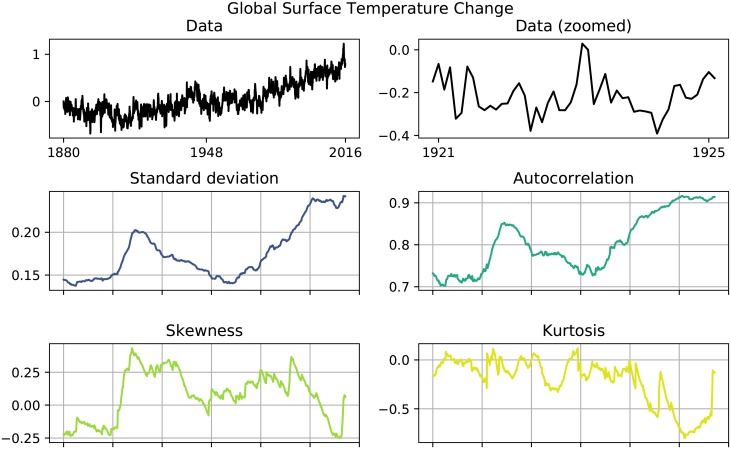
Global surface temperature change. Both standard deviation and autocorrelation increase, which can be interpreted as an EWS.

Results of the EWS analysis are presented in Fig 7. As expected, we detect a rise in autocorrelation and standard deviation. However, we suspect that this rise should not be interpreted as an EWS, but rather as a general feature of a system resembling the abstract system QG. To check this hypothesis, we detrend the data using moving averages and repeat the EWS analysis.

The global surface temperature change shows exactly the same behavior as the abstract system QG: After detrending the increase in autocorrelation and standard deviation vanishes and we are unable to detect any EWS. The previously detected EWS was indeed a false positive, meaning that our insights from abstract, generic systems can be translated to real-world systems.

## Discussion

In this study we were able to show that many different systems are prone to false positives in EWS analysis, when investigating raw data. Large classes of systems, among them exponentially growing systems, systems featuring logistic growth and systems experiencing logarithmic decay, can inherently show an increase in standard deviation as well as autocorrelation at lag 1, which is usually interpreted as an EWS [11]. This means, that especially in systems that resemble such behavior, a traditional EWS analysis is not viable, since it is susceptible to false positives.

We also investigated strategies to circumvent such false positives. While scaling of the analyzed data was not able to remove the false positives, detrending of the data was successful. When looking at the residuals, all false positives disappeared in the investigated systems. All investigated methods of detrending (local first-order polynomial fit, global higher-order polynomial fit and moving averages) lead to the same result. However, using moving averages has some advantages, since it is numerically more efficient and its effect is independent on the chosen window size. Application to real world data of the global surface temperature change substantiated that these findings can be translated from generic to real-world systems. However, there are also some limitations to our study. In order to investigate many different systems within the same framework we rely on data from a model. Although this study suggests that this computer-generated data can be used to gain insight to the properties of data measured in the real world, as substantiated by the analysis of the global temperature change data, this is of course no proof that all the understanding we gain from simulated data can be directly translated to real-life data. Also not all real-life systems can be approximated by the 12 abstract systems we investigated. Systems with more complicated behavior or fluctuations that are neither absolute nor relative are not within the scope of this investigation. Nevertheless, the studied systems can always serve as a starting point for a more thorough analysis of an arbitrary system.

This study also raises an interesting question: How does detrending affect true positives? Is it possible to have systems with critical transitions that show EWS only in undetrended data? A lot of recent studies use detrending for EWS analysis and to our knowledge none of them report on such a phenomenon, where detrending removes the EWS [38–43]. However, to further substantiate this, one would need to rigorously investigate time series from systems with guaranteed critical transitions that show no EWS after detrending and see if they show EWS if no form of detrending is used. Since studies that fail to find EWS in systems are rarely published because of publication bias, it might be necessary to collect or simulate such data in order to perform such a study, which would be an interesting direction for further investigations.

In conclusion, we showed that systematic false positives in EWS analysis are quite common in many systems which makes it difficult to make accurate predictions about critical transitions in such cases. In order to remove those false positives, data must always be detrended and the EWS analysis performed on the resulting residuals. That way, also systems prone to systematic false positives can be investigated with the usual EWS analysis techniques.

### Appendix A—Numerical details of the investigated systems

The time series *x*_*i*_ of length *N* that were analyzed for EWS were calculated from the following equations:

$$x_{i}=\frac{i}{N}$$ (LG)

$$x_{i}={\left(\frac{i}{N}\right)}^{2}$$ (QG)

$$\begin{array}{c} x_{i}=\exp(\frac{i}{N}-1) \end{array}$$ (EG)

$$\begin{array}{c} x_{i}=\ln(1+\frac{i}{N}) \end{array}$$ (LNG)

$$\begin{array}{c} x_{i}=\sin(\frac{i\;\pi }{2\;N}) \end{array}$$ (TG)

$$\begin{array}{c} x_{i}=x_{i-1}+\alpha \;x_{i-1}\;\left(1-x_{i-1}\right),\;\;\;x_{1}=0.01,\;\;\;\alpha =5/N \end{array}$$ (LOG)

$$\begin{array}{c} x_{i}=1-\frac{i}{N} \end{array}$$ (LD)

$$\begin{array}{c} x_{i}=1-{\left(\frac{i}{N}\right)}^{2} \end{array}$$ (QD)

$$\begin{array}{c} x_{i}=\exp(\frac{-i}{N}-1) \end{array}$$ (ED)

$$\begin{array}{c} x_{i}=\ln(2-\frac{i}{N}) \end{array}$$ (LND)

$$\begin{array}{c} x_{i}=\cos(\frac{i\;\pi }{2\;N}) \end{array}$$ (TD)

$$\begin{array}{c} x_{i}=x_{i-1}-\alpha \;x_{i-1}\;\left(1-x_{i-1}\right),\;\;\;x_{1}=0.99,\;\;\;\alpha =5/N \end{array}$$ (LOD)

For absolute noise Gaussian-distributed random numbers with mean 0 and standard deviation 0.01 were added to each data point. For relative noise each data point was multiplied by a Gaussian-distributed random number with mean 1 and standard deviation 0.01.
